# Supplementary material for: Hierarchical self-assembly of simple hard polyhedra into complex mesophases
Source: Nat Commun. 2025 Dec 4;16:10887. doi: 10.1038/s41467-025-65891-w (PMC12678431; doi:10.1038/s41467-025-65891-w)
Supplement: Supplementary file 1 — Supplementary Information [file 41467_2025_65891_MOESM1_ESM.pdf]

Supplementary Information
Hierarchical Self-assembly of Simple Hard Polyhedra into Complex Mesophases

Rodolfo Subert[1] and Marjolein Dijkstra[1,2*]

[1] *Soft Condensed Matter & Biophysics, Debye Institute for Nanomaterials Science, Utrecht University, Princetonplein 5, 3584 CC Utrecht, The Netherlands.* and

[2] *International Institute for Sustainability with Knotted Chiral Meta Matter (SKCM^2), Hiroshima University, 1-3-1 Kagamiyama, Higashi-Hiroshima, Hiroshima, 739-8526, Japan*

* m.dijkstra@uu.nl

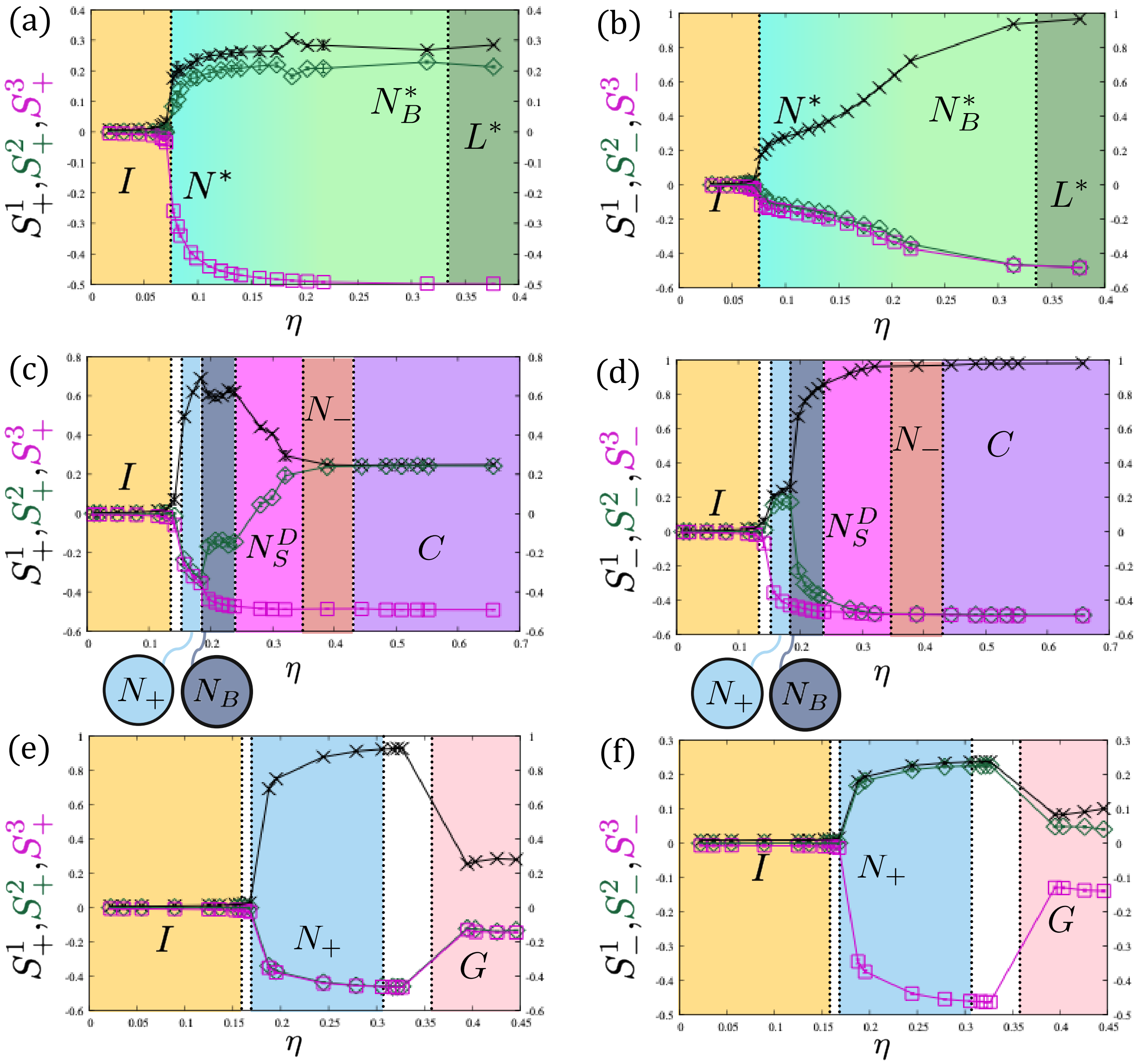

Supplementary Fig. 1. The full eigenvalue signatures, S^1_+, S^2_+, S^3_+ and S^1_-, S^2_-, and S^3_-, of the nematic order parameter tensors for the long particle axes $\hat{\mathbf{l}}$ and the short particle axes $\hat{\mathbf{t}}$, respectively, as a function of packing fraction η as obtained from simulations of varying hard, distorted tetrahedral particles. The panels correspond to particles with a length-to-width ratio L/W and width-to-thickness ratio W/T values of $L/W, W/T = 5, 4$ (a,b); 2.5, 4 (c,d); and 2.5, 2 (e,f).

I. ORDER PARAMETERS

In the main text, we distinguish the various nematic phases by analyzing two nematic order parameters, S_+ and S_-, which correspond to the largest eigenvalues of the 3×3 nematic order

parameter tensor $\mathcal{Q} = \left\langle \frac{3}{2}\hat{\boldsymbol{a}} \otimes \hat{\boldsymbol{a}} - \frac{1}{2}\mathbb{I} \right\rangle$, where $\hat{\boldsymbol{a}}$ represents either the long particle axis $\hat{\boldsymbol{l}}$ for S_+ or the short particle axis $\hat{\boldsymbol{t}}$ for S_-. While S_+ and S_- provide a straightforward and interpretable means of distinguishing nematic phases, their applicability is constrained by the difficulty of defining a consistent local frame of reference across all model particles. To achieve a more robust characterization of the orientational distributions within different nematic phases, we extend our analysis by examining the full eigenvalue signatures of the two nematic order parameter tensors. These signatures are compared with the theoretical expectation values of the nematic order parameter tensors for well-defined director field profiles corresponding to known nematic phases, including prolate and oblate uniaxial nematics, biaxial, chiral, chiral biaxial, and splay nematic phases.

For a given director field profile defined by the orientation dependent triple $\hat{\boldsymbol{n}}_{\hat{\boldsymbol{l}}}(\Omega)$, $\hat{\boldsymbol{n}}_{\hat{\boldsymbol{t}}}(\Omega)$ and $\hat{\boldsymbol{n}}_{\hat{\boldsymbol{w}}}(\Omega) = \hat{\boldsymbol{n}}_{\hat{\boldsymbol{l}}}(\Omega) \times \hat{\boldsymbol{n}}_{\hat{\boldsymbol{t}}}(\Omega)$, the nematic order parameter tensors are given by the integral averages

$$Q_{\pm_{\alpha\beta}} = \frac{1}{|\Omega|} \int_{\Omega} d\Omega \left(\frac{3}{2} \hat{\boldsymbol{a}}_{\alpha}(\Omega) \hat{\boldsymbol{a}}_{\beta}(\Omega) - \frac{1}{2}\delta_{\alpha\beta} \right), \tag{1}$$

where we use $\hat{\boldsymbol{a}}(\Omega) = \hat{\boldsymbol{n}}_{\hat{\boldsymbol{l}}}(\Omega)$ for $\mathbf{Q}_+$, and $\hat{\boldsymbol{a}}(\Omega) = \hat{\boldsymbol{n}}_{\hat{\boldsymbol{t}}}(\Omega)$ for $\mathbf{Q}_-$. Here, Ω represents one or more angular variables, which may or may not explicitly depend on a spatial coordinate describing a modulation, such as a one-dimensional periodic variation in cholesteric phases or the uniform orientation found in oblate nematic phases. The term $|\Omega|$ denotes the appropriate normalization factor.

A. Prolate Uniaxial Nematic N_+ Phase

In a prolate uniaxial nematic N_+ phase, we assume that the primary director $\hat{\boldsymbol{n}}_{\hat{\boldsymbol{l}}}$ is always aligned with the $\hat{z}$-axis, while the secondary directors are uniformly distributed in the plane perpendicular to $\hat{\boldsymbol{n}}_{\hat{\boldsymbol{l}}}$:

$$\hat{\boldsymbol{n}}_{\hat{\boldsymbol{l}}} = \begin{pmatrix} 0 \\ 0 \\ 1 \end{pmatrix}, \quad \hat{\boldsymbol{n}}_{\hat{\boldsymbol{t}}} = \begin{pmatrix} \sin(\phi) \\ \cos(\phi) \\ 0 \end{pmatrix}, \quad \hat{\boldsymbol{n}}_{\hat{\boldsymbol{w}}} = \begin{pmatrix} -\cos(\phi) \\ \sin(\phi) \\ 0 \end{pmatrix}. \tag{2}$$

The corresponding nematic order parameter tensors are given by

$$\mathbf{Q}_{+} = \begin{pmatrix} -\frac{1}{2} & 0 & 0 \\ 0 & -\frac{1}{2} & 0 \\ 0 & 0 & 1 \end{pmatrix}, \quad \mathbf{Q}_{-} = \begin{pmatrix} \frac{1}{4} & 0 & 0 \\ 0 & \frac{1}{4} & 0 \\ 0 & 0 & -\frac{1}{2} \end{pmatrix}. \tag{3}$$

Here, the eigenvalue signature of $\{1/4, 1/4, -1/2\}$ of $\mathbf{Q}_{-}$ corresponds to an orientational average over a cylindrical distribution. Consistent with typical configurations observed in simulations, we observe that the nematic order parameter values in the light blue regions of SFig1.c, d, e, and f match this signature.

B. Oblate Uniaxial Nematic N_{-} Phase

In an oblate uniaxial nematic N_{-} phase, we assume that the primary director $\hat{\boldsymbol{n}}_{\hat{\boldsymbol{t}}}$ is always aligned with the $\hat{z}$-axis, while the secondary directors $\hat{\boldsymbol{n}}_{\hat{\boldsymbol{l}}}$ and $\hat{\boldsymbol{n}}_{\hat{\boldsymbol{w}}}$ are uniformly distributed in the plane perpendicular to $\hat{\mathbf{t}}$:

$$\hat{\boldsymbol{n}}_{\hat{\boldsymbol{l}}} = \begin{pmatrix} \sin(\phi) \\ \cos(\phi) \\ 0 \end{pmatrix}, \quad \hat{\boldsymbol{n}}_{\hat{\boldsymbol{t}}} = \begin{pmatrix} 0 \\ 0 \\ 1 \end{pmatrix}, \quad \hat{\boldsymbol{n}}_{\hat{\boldsymbol{w}}} = \begin{pmatrix} -\cos(\phi) \\ \sin(\phi) \\ 0 \end{pmatrix}. \tag{4}$$

The associated nematic order parameter tensors are

$$\mathbf{Q}_{+} = \begin{pmatrix} \frac{1}{4} & 0 & 0 \\ 0 & \frac{1}{4} & 0 \\ 0 & 0 & -\frac{1}{2} \end{pmatrix}, \quad \mathbf{Q}_{-} = \begin{pmatrix} -\frac{1}{2} & 0 & 0 \\ 0 & -\frac{1}{2} & 0 \\ 0 & 0 & 1 \end{pmatrix}. \tag{5}$$

As seen from the eigenvalue structures of $\mathbf{Q}_{+}$ and $\mathbf{Q}_{-}$, the transition of orientational ordering from the long particle axis in prolate nematics to the short particle axis in oblate nematics is reflected in the interchange of eigenvalues between the two nematic order parameter tensors. In agreement with our simulations, oblate nematic ordering is observed in the brown high-density regions of Supplementary Fig.1 c and d.

C. Biaxial Nematic N_B Phase

In a biaxial nematic N_B phase, we assume that the three directors are always aligned along the three Cartesian axes:

$$\hat{\boldsymbol{n}}_{\hat{\boldsymbol{l}}} = \begin{pmatrix} 0 \\ 0 \\ 1 \end{pmatrix}, \quad \hat{\boldsymbol{n}}_{\hat{\boldsymbol{t}}} = \begin{pmatrix} 1 \\ 0 \\ 0 \end{pmatrix}, \quad \hat{\boldsymbol{n}}_{\hat{\boldsymbol{w}}} = \begin{pmatrix} 0 \\ 1 \\ 0 \end{pmatrix}. \tag{6}$$

The corresponding nematic order parameter tensors are given by

$$\mathbf{Q}_{+} = \begin{pmatrix} -\frac{1}{2} & 0 & 0 \\ 0 & -\frac{1}{2} & 0 \\ 0 & 0 & 1 \end{pmatrix}, \quad \mathbf{Q}_{-} = \begin{pmatrix} 1 & 0 & 0 \\ 0 & -\frac{1}{2} & 0 \\ 0 & 0 & -\frac{1}{2} \end{pmatrix}. \tag{7}$$

According to the eigenvalue signatures, biaxial order is observed in the gray regions of Supplementary Fig.1 c and d.

D. Cholesteric N^* Phases

In a cholesteric liquid crystal, the orientation of the primary director $\hat{\boldsymbol{n}}_{\hat{\boldsymbol{l}}}$ varies continuously along the chiral axis according to $\phi = qz$, where $P = 2\pi/q$ represents the cholesteric pitch. Additionally, we assume that the other two directors, $\hat{\boldsymbol{n}}_{\hat{\boldsymbol{t}}}$ and $\hat{\boldsymbol{n}}_{\hat{\boldsymbol{w}}}$, remain perpendicular to $\hat{\boldsymbol{n}}_{\hat{\boldsymbol{l}}}$ and are randomly distributed around it

$$\hat{\boldsymbol{n}}_{\hat{\boldsymbol{l}}} = \begin{pmatrix} \sin(qz) \\ \cos(qz) \\ 0 \end{pmatrix}, \quad \hat{\boldsymbol{n}}_{\hat{\boldsymbol{t}}} = \begin{pmatrix} \cos(qz)\sin(\theta) \\ -\sin(qz)\sin(\theta) \\ \cos(\theta) \end{pmatrix}, \quad \hat{\boldsymbol{n}}_{\hat{\boldsymbol{w}}} = \begin{pmatrix} -\cos(qz)\cos(\theta) \\ \sin(qz)\cos(\theta) \\ \sin(\theta) \end{pmatrix}. \tag{8}$$

The corresponding nematic order parameter tensors are

$$\mathbf{Q}_{+} = \begin{pmatrix} \frac{1}{4} & 0 & 0 \\ 0 & \frac{1}{4} & 0 \\ 0 & 0 & -\frac{1}{2} \end{pmatrix}, \quad \mathbf{Q}_{-} = \begin{pmatrix} -\frac{1}{8} & 0 & 0 \\ 0 & -\frac{1}{8} & 0 \\ 0 & 0 & \frac{1}{4} \end{pmatrix}. \tag{9}$$

As expected, the eigenvalue signature of $\mathbf{Q}_{+}$ aligns with that of $\mathbf{Q}_{+}$ for oblate uniaxial nematics or $\mathbf{Q}_{-}$ for prolate uniaxial nematics, reflecting the uniaxial cylindrical distribution of $\hat{\boldsymbol{n}}_{\hat{\boldsymbol{l}}}$. We observe this signature in the green-blue N* regions of SFig1. a and b.

E. Cholesteric Biaxial N_B^* Phases

In a cholesteric biaxial N_B^* phase, we assume that the secondary director $\hat{\boldsymbol{n}}_{\hat{\boldsymbol{t}}}$ is aligned with the $\hat{z}$-axis

$$\hat{\boldsymbol{n}}_{\hat{\boldsymbol{l}}} = \begin{pmatrix} \sin(qz) \\ \cos(qz) \\ 0 \end{pmatrix}, \quad \hat{\boldsymbol{n}}_{\hat{\boldsymbol{t}}} = \begin{pmatrix} 0 \\ 0 \\ 1 \end{pmatrix}, \quad \hat{\boldsymbol{n}}_{\hat{\boldsymbol{w}}} = \begin{pmatrix} \cos(qz) \\ -\sin(qz) \\ 0 \end{pmatrix}, \tag{10}$$

and the corresponding nematic order parameters are

$$\mathbf{Q}_+ = \begin{pmatrix} \frac{1}{4} & 0 & 0 \\ 0 & \frac{1}{4} & 0 \\ 0 & 0 & -\frac{1}{2} \end{pmatrix} \quad \mathbf{Q}_- = \begin{pmatrix} -\frac{1}{2} & 0 & 0 \\ 0 & -\frac{1}{2} & 0 \\ 0 & 0 & 1 \end{pmatrix} \tag{11}$$

The cholesteric biaxial phase is simpler. It shares the same distribution of a cholesteric in $\mathbf{Q}_+$, but uniaxial ordering in $\mathbf{Q}_-$. We observe that the cholesteric phase in Supplementary Fig.1 a and b slowly transitions to a biaxial cholesteric phase.

F. Splay Nematic and Splay Biaxial Nematic Phases

We assume the splay nematic phase is characterized by a periodic modulation of the primary director $\hat{\boldsymbol{n}}_{\hat{\boldsymbol{l}}}$ along the x-direction with a pitch length $P = 2\pi/q$, while $\hat{\boldsymbol{n}}_{\hat{\boldsymbol{t}}}$ remains aligned with the z-axis.

$$\hat{\boldsymbol{n}}_{\hat{\boldsymbol{l}}} = \begin{pmatrix} \sin(\theta_0 \sin(qx)) \\ \cos(\theta_0 \sin(qx)) \\ 0 \end{pmatrix}, \quad \hat{\boldsymbol{n}}_{\hat{\boldsymbol{t}}} = \begin{pmatrix} 0 \\ 0 \\ 1 \end{pmatrix}, \quad \hat{\boldsymbol{n}}_{\hat{\boldsymbol{w}}} = \begin{pmatrix} \cos(\theta_0 \sin(qx)) \\ -\sin(\theta_0 \sin(qx)) \\ 0 \end{pmatrix}. \tag{12}$$

The corresponding nematic order parameters are

$$\mathbf{Q}_+ = \begin{pmatrix} \frac{1}{4} - \frac{3}{4}J_0(2\theta_0) & 0 & 0 \\ 0 & \frac{1}{4} + \frac{3}{4}J_0(2\theta_0) & 0 \\ 0 & 0 & -\frac{1}{2} \end{pmatrix} \quad \mathbf{Q}_- = \begin{pmatrix} -\frac{1}{2} & 0 & 0 \\ 0 & -\frac{1}{2} & 0 \\ 0 & 0 & 1 \end{pmatrix}, \tag{13}$$

where J_0 represents the zeroth-order Bessel function of the first kind. We recover the nematic order parameters for the biaxial nematic phase when $\theta_0 = 0$. Additionally, the nematic order parameters evolve towards those of an oblate nematic phase upon increasing θ_0. Interestingly,

in Supplementary Fig.1 c and d, we observe that the nematic order parameter values in the pink region, where the system transitions from a biaxial nematic phase to an oblate nematic phase, exhibit this signature. Visual inspection of the configurations reveals no periodic splay modulation in the nematic director fields $\hat{\boldsymbol{n}}_{\hat{l}}$ and $\hat{\boldsymbol{n}}_{\hat{w}}$, but instead show local splay domains randomly distributed in the xy-plane. The splay angle θ_0 increases with density upon transitioning from the biaxial nematic phase to the oblate nematic phase. We refer to this state as the disordered splay nematic N_S^D phase. The nematic eigenvalue signatures of the N_S^D phase are identical to those of the periodically modulated splay nematic phase. We note that an arbitrary $f(qx)$ can be used instead of $\sin(qx)$, allowing the oblate nematic phase to be recovered exactly when

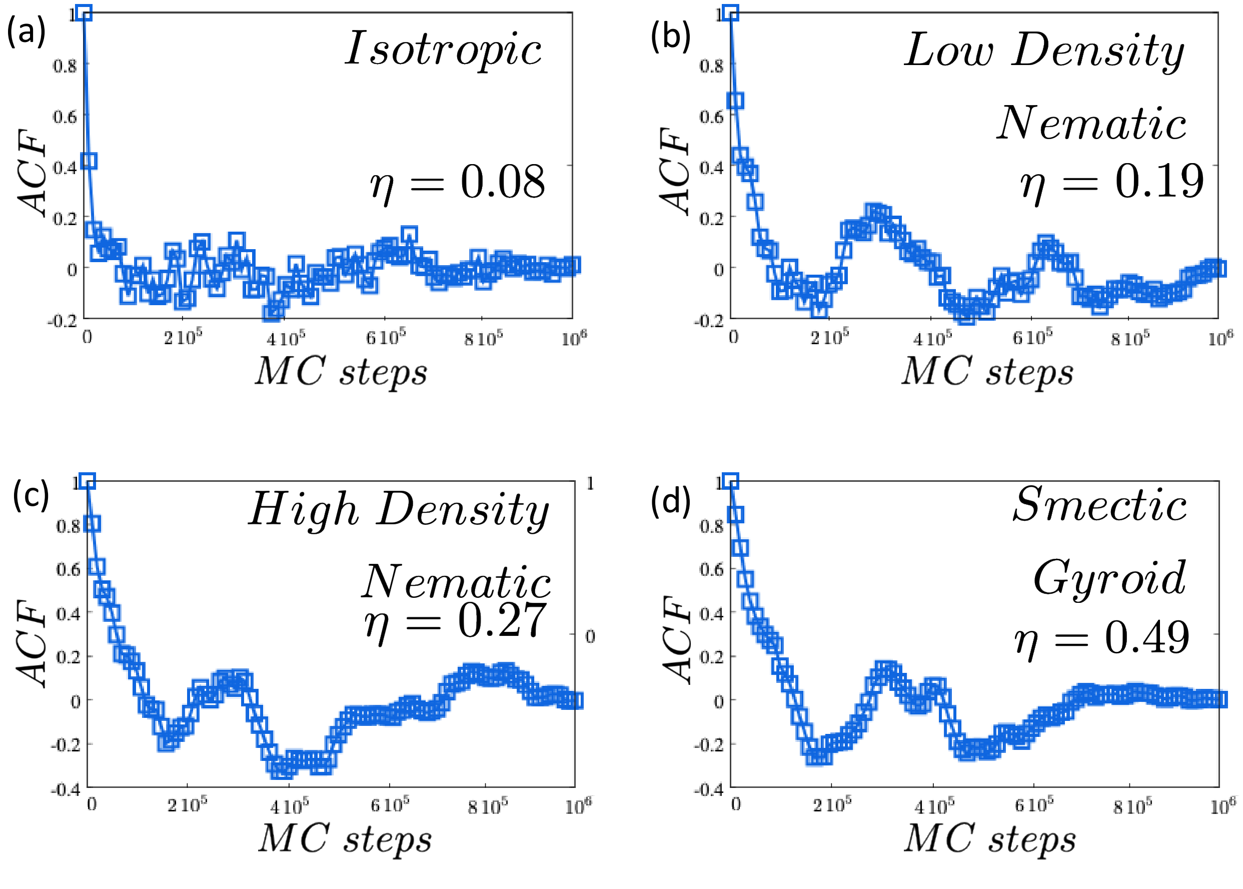

Supplementary Fig. 2. Density autocorrelation functions for moderately anisotropic particles, computed over 10^6 steps from equilibrated configurations at four representative densities: (a) isotropic phase at $\eta = 0.08$, (b) low-density prolate uniaxial nematic at $\eta = 0.19$, (c) high-density prolate uniaxial nematic at $\eta = 0.27$, and (d) gyroid phase at $\eta = 0.49$. The results show the expected increase in correlation time with density, reaching its maximum in the gyroid phase.

$\theta_0 = \pi/2$.

II. MONTE CARLO SIMULATION DETAILS

To investigate the self-assembly behavior of the hard distorted tetrahedra presented in the main text, we performed Monte Carlo simulations using the HOOMD-blue package[1]. Simulations of these systems demand carefully designed initialization, compression, and equilibration protocols—especially at high densities, in phases with narrow stability windows, and under

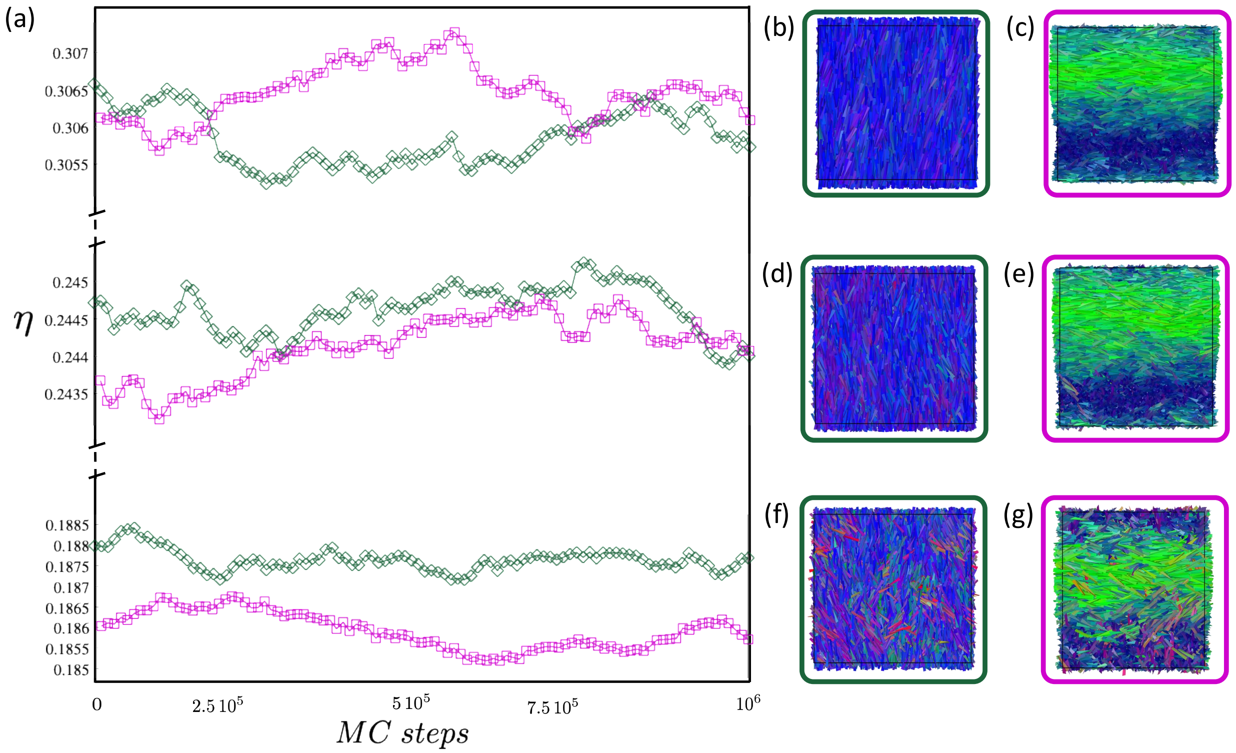

Supplementary Fig. 3. (a) Packing fraction η as a function of Monte Carlo (MC) steps for the uniaxial nematic (green diamonds) and cholesteric (magenta squares) phases at three different pressures $\beta P v = 3.3$, $\beta P v = 2.0$ and $\beta P v = 1.25$ shown after equilibrium is reached. The uniaxial nematic phase systematically exhibits a slightly higher equilibrium packing fraction than the cholesteric phase, with differences appearing at the third or fourth decimal place. This suggests greater stability for the uniaxial nematic phase while also explaining the spontaneous nucleation of cholesteric phases. (b,d,f) Representative configurations of the uniaxial nematic phase at the three considered pressures. (c,e,g) Representative configurations of the cholesteric phase at the same pressures.

conditions susceptible to metastable states.

All simulations were performed in the NPT ensemble by independently proposing anisotropic volume moves along one of the three axes, with acceptance probability

$$P_{acc} = \min\left[1,\ \exp\left(\beta P(V - V') - N \ln \frac{V'}{V}\right)\right],$$

where V and V' are the volumes before and after the proposed move, respectively. Moves that would result in particle overlaps were automatically rejected. Particles were allowed to both translate and rotate, with acceptance ratios maintained between 40–60%. Each Monte Carlo step consisted of one attempted translation, one attempted rotation per particle, and a single attempted volume move.

To reduce computational cost during parameter exploration and to evaluate potential finite-size effects, we first performed exploratory runs with 3,000 particles. We then increased the system size to 24,000 particles, focusing on a narrower set of particle shapes. In all cases, compressions were initialized from a low-density configuration, with particles placed on a grid at one particle per $8L^3$ units of volume. Compression across the isotropic to nematic phase transition proved to be the most delicate part of the protocol, requiring slow adjustment to maintain the target acceptance ratios.

Depending on the target phase and its density, the equilibration stage required anywhere from about 10^6 steps (sufficient to equilibrate low-density phases) up to roughly 2×10^7 cumulative steps (for higher-density smectic phases). For the latter, equilibration was monitored in batches of 10^6 steps. Once a state point was deemed equilibrated, we performed an additional production run of 10^6–2×10^6 steps to measure observables. These choices were guided by the packing fraction auto-correlation function

$$C(t) = \frac{\langle(\langle \eta(0) - \langle\eta\rangle\rangle)(\eta(t) - \langle\eta\rangle\rangle)\rangle}{\langle(\eta(0) - \langle\eta\rangle)^2\rangle},$$

where $\langle \dots \rangle$ denotes the ensemble average. Correlation times were roughly estimated as the time at which $C(t)$ first crosses zero. In the isotropic phase, correlation times were short ($\sim 10^4$ steps, Supplementary Fig. 2a), increased to $\sim 5 \times 10^4$ steps in the nematic regime (Supplementary Fig. 2b), and reached up to $\sim 10^5$ steps in denser nematic phases (Supplementary Fig. 2c). The longest correlation times occured in the gyroid phase, peaking at $\sim 2 \times 10^5$ steps (Supplementary Fig. 2c) at density $\eta = 0.49$. Our protocol therefore ensured equilibration, as run lengths exceeded correlation times in all cases. This was further confirmed by monitoring the packing fraction, which fluctuates only at the fourth decimal place over 10^6 steps (Supplementary Fig.3).

While simulations generally proceeded smoothly, we observed one notable case of kinetic trapping: moderately anisotropic particles exhibited two competing metastable states. This case required additional analysis to assess phase stability, as described in the following section.

III. METASTABLE CHIRAL PHASES

Moderately anisotropic particles with aspect ratios $L/W = 5$ and $W/T = 1$ can assemble into either a uniaxial nematic N or a cholesteric N* phase with similar likelihood upon compression from the isotropic I phase, as shown in Supplementary Fig.2. A careful comparison of the packing fractions of these two phases across simulations at different pressures $\beta P v = 3.3$, $\beta P v = 2.0$ and $\beta P v = 1.25$, spanning the whole nematic region of stability, reveals that the uniaxial nematic phase is consistently slightly denser, differing by the third decimal place, supporting that the uniaxial N phase is more stable. This behavior highlights the subtle role of saddle-splay contributions in stabilizing chiral textures. When melting from the gyroid phase, only the uniaxial nematic phase is observed, further corroborating the stability of the uniaxial nematic phase with respect to the cholesteric phase.

IV. ELASTIC CONSTANTS

Similar to the nematic order parameters, liquid crystal elastic constants are affected by the choice of a particle reference frame and the definition of the particle main axis, particularly, in phase sequences where the symmetry axis changes. This issue is thoroughly discussed in Ref. [2], where the splay-bend nematic N_{SB} phase is compared with the splay nematic N_S phase. To avoid ambiguities related to the reference frame and to verify whether the main trends and expectations hold, we compute the uniaxial elastic constants relative to the long particle axis $\hat{\mathbf{l}}$ using the standard second-virial approximation [3, 4]

$$\beta D K_1 = -\frac{a(\eta)n^2}{2}\int d\mathbf{r}d\hat{\mathbf{l}}_1 d\hat{\mathbf{l}}_2 c^{(2)}(\mathbf{r},\hat{\mathbf{l}}_1,\hat{\mathbf{l}}_2)x^2 l_{1,x} l_{2,x}\psi'(\hat{\mathbf{l}}_1)\psi'(\hat{\mathbf{l}}_2), \tag{14}$$

$$\beta D K_2 = -\frac{a(\eta)n^2}{2}\int d\mathbf{r}d\hat{\mathbf{l}}_1 d\hat{\mathbf{l}}_2 c^{(2)}(\mathbf{r},\hat{\mathbf{l}}_1,\hat{\mathbf{l}}_2)x^2 l_{1,y} l_{2,y}\psi'(\hat{\mathbf{l}}_1)\psi'(\hat{\mathbf{l}}_2), \tag{15}$$

$$\beta D K_3 = -\frac{a(\eta)n^2}{2}\int d\mathbf{r}d\hat{\mathbf{l}}_1 d\hat{\mathbf{l}}_2 c^{(2)}(\mathbf{r},\hat{\mathbf{l}}_1,\hat{\mathbf{l}}_2)z^2 l_{1,x} l_{2,x}\psi'(\hat{\mathbf{l}}_1)\psi'(\hat{\mathbf{l}}_2), \tag{16}$$

$$K_{24} = \frac{1}{2}(K_2 - K_1). \tag{17}$$

Here x and z represent the respective projections of the particle center-of-mass distance $\mathbf{r}$, $\hat{\mathbf{l}}_i$ denotes the particle orientation, $c^{(2)}(\mathbf{r}, \hat{\mathbf{l}}_1, \hat{\mathbf{l}}_2)$ is the direct correlation function, which reduces to the Mayer function for hard particles. The factor $a(\eta)$ is the Lee density-dependent correction factor [5] for the pair correlation function, and $\psi(\hat{\mathbf{l}}_i)$ is the orientational distribution function normalized as $2\pi \int d\hat{\mathbf{l}}_i \psi(\hat{\mathbf{l}}_i) = 1$, for which we will use the Gaussian ansatz

$$\psi(\hat{\mathbf{l}}_i) = \frac{G^2(\eta)c_0^2}{Z\pi^2} \exp(-\frac{2}{\pi}G^2(\eta)c_0^2\theta^2), \tag{18}$$

where $G(\eta)$ is the Parsons-Lee [6, 7] correction factor for the orientational distribution function of finite length particles, θ is the angle between $\hat{\mathbf{l}}$ and the z-axis, and D represents the effective diameter of a distorted tetrahedron, given by $D = (T + W)/2$, the average of the two diameters. We use this effective diameter to map the Onsager dimensionless concentration $c_0 = \pi L^2 D n/4$ for spherocylinders to our system, where n is the number density. Note that the Gaussian ansatz for the orientational distribution function assumes a uniaxial distribution, which is valid only for the uniaxial nematic phase.

Numerically we proceed as follows: First, we generate a pair of particles, one with its center of mass at the origin and the other within a sphere of radius R from the origin, where R is the maximum distance at which two distorted tetrahedra can overlap. Both particles have independently and uniformly drawn orientation.

To gain insights in the system behaviour, we compare the phase diagram obtained from simulations to the splay K_1 and twist K_2 elastic constants, their ratio K_1/K_2, and their difference corresponding to $K_{24} = (K_1 - K_2)/2$, respectively, in Supplementary Fig. 3 a-c and d-f. While a full analysis of the elastic constants with respect to all particle axes and orientational distribution functions of the various nematic phases would require a more detailed investigation, the simple calculation presented here provides valuable insights. Since we measure the elastic constants relative to the $\hat{\mathbf{l}}$ axis and assume local uniaxiality, their magnitudes reflect the stiffness of local modulations concerning local alignment of particles along $\hat{\mathbf{l}}$. In Supplementary Fig.3, we present the elastic constants for rod-like (a,b), disk-like (c,d), and moderately anisotropic particles (e,f) along with the phase sequences observed in simulations, indicated by the background.

In the left column, we present the splay K_1 and twist K_2 elastic constants. Notably, they both exhibit similar and distinct variations at the phase transition densities, capturing both the isotropic-nematic I-N and nematic-smectic N-Sm phase transitions. We omit K_3 from the plots, as it is consistently several orders of magnitude larger, confirming that twist and splay

deformations are significantly softer. It is also important to note that the splay and twist modes in this context correspond to double-splay and double-twist deformations, which, while soft, remain positive–consistent with the absence of such local modulations in our simulations. However, as shown in Supplementary Fig.3b,d, and f, the saddle-splay elastic constant, calculated using Poniewierski's formulation, is consistently negative at the isotropic-nematic transition for all three particle types. This indicates a strong propensity for Δ-driven modulations and highlights its crucial role in stabilising both the cholesteric and disordered splay nematic phase. While this calculation successfully identifies the smectic regime, it provides limited insight into the precise nature of the emerging structures. Additionally, the splay elastic constant remains consistently lower than the twist elastic constant ($K_1 < K_2$). This contrasts with the predictions of Ref. [8], which suggest that distorted tetrahedra stabilize cholesteric phases when $K_2 < K_1$. This discrepancy may stem from calculating the elastic constants relative to the long particle axis $\hat{\mathbf{l}}$.

In summary, although a direct comparison with Selinger's framework [9] remains challenging and further analysis is required to fully characterize the resulting structures, our simple calculation successfully captures the I-N and N-Sm phase transition densities and highlights the critical role of K_{24} in stabilising the observed nematic phases.

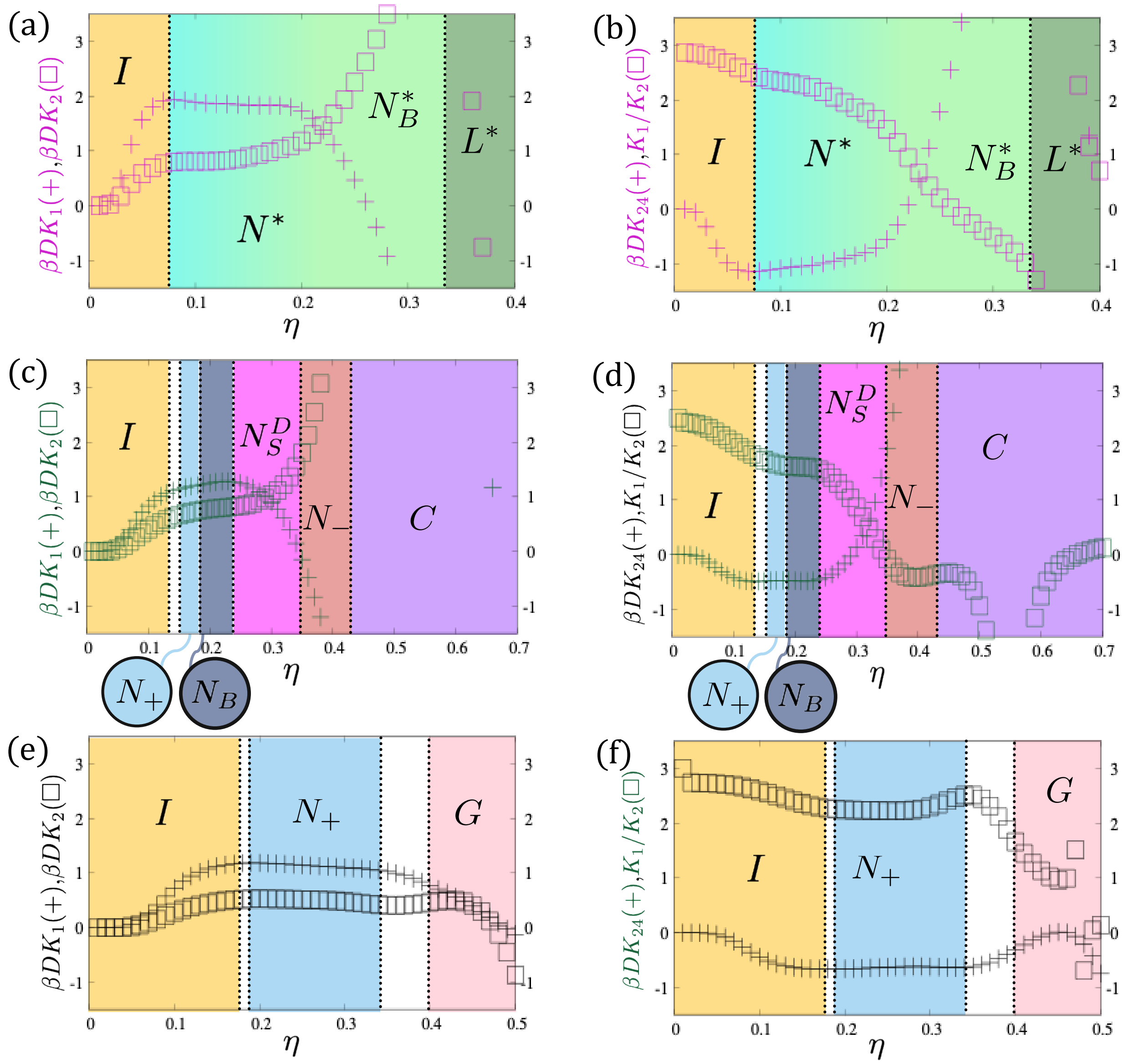

Supplementary Fig. 4. Comparison of splay K_1 and twist K_2 elastic constants (left column) with the saddle-splay $K_{24} = (K_1 - K_2)/2$ constant and the splay-twist K_1/K_2 ratio (right column) across the simulated phase diagram for rod-like, disk-like, and moderately anisotropic particles in Supplementary Fig.3a,b, c,d, and e,f, respectively. The results demonstrate a strong correlation between the elastic constants and the phase transitions observed in simulations, highlighting the role of K_{24} in stabilising the observed nematic textures.

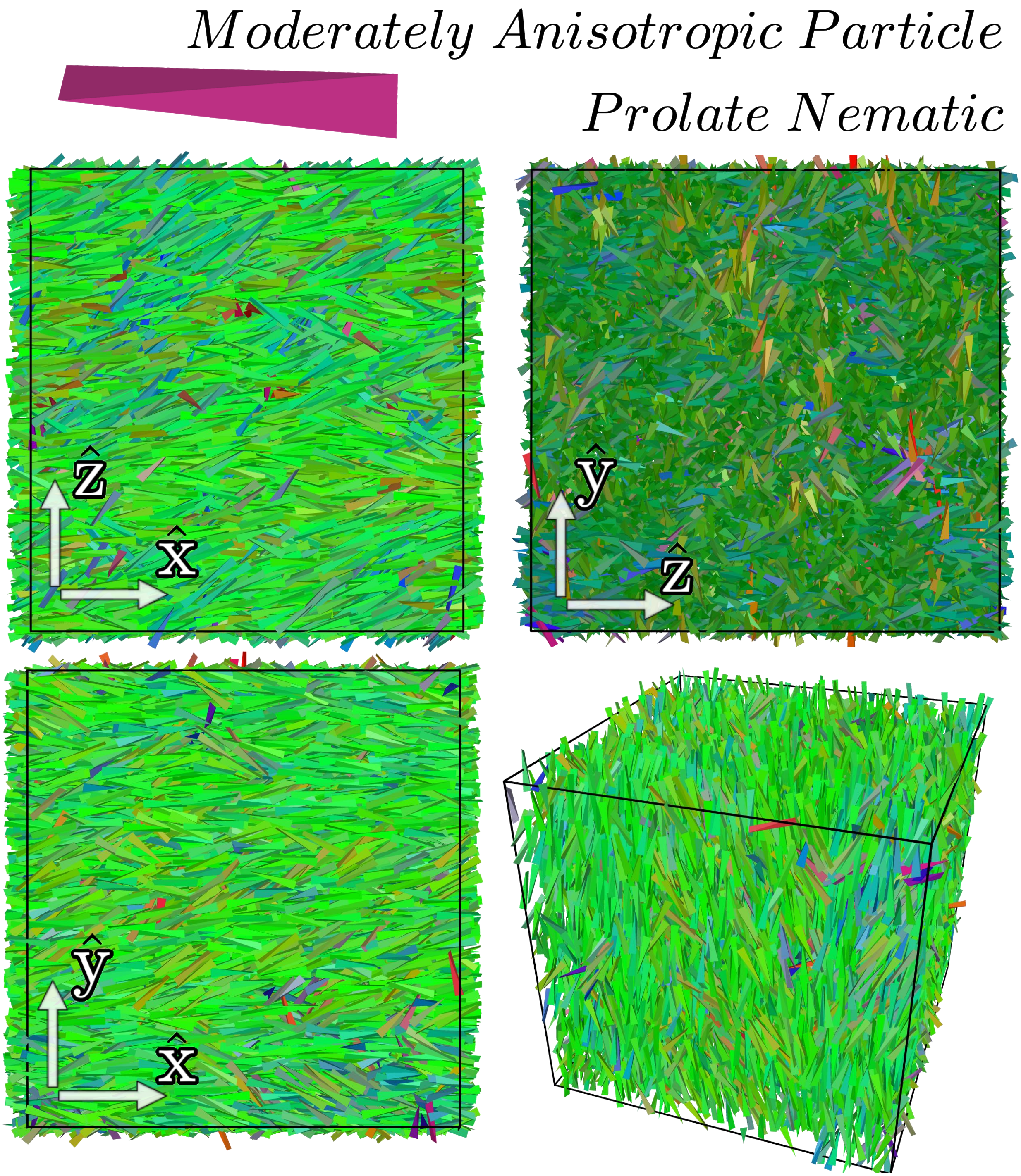

Supplementary Fig. 5. Typical configuration of a system of moderately anisotropic particles in the prolate nematic phase. The same configuration is shown along the three coordinate axes and in perspective. Particles are colored according to their orientation: green for the x axis, red for the y axis, and blue for the z axis.

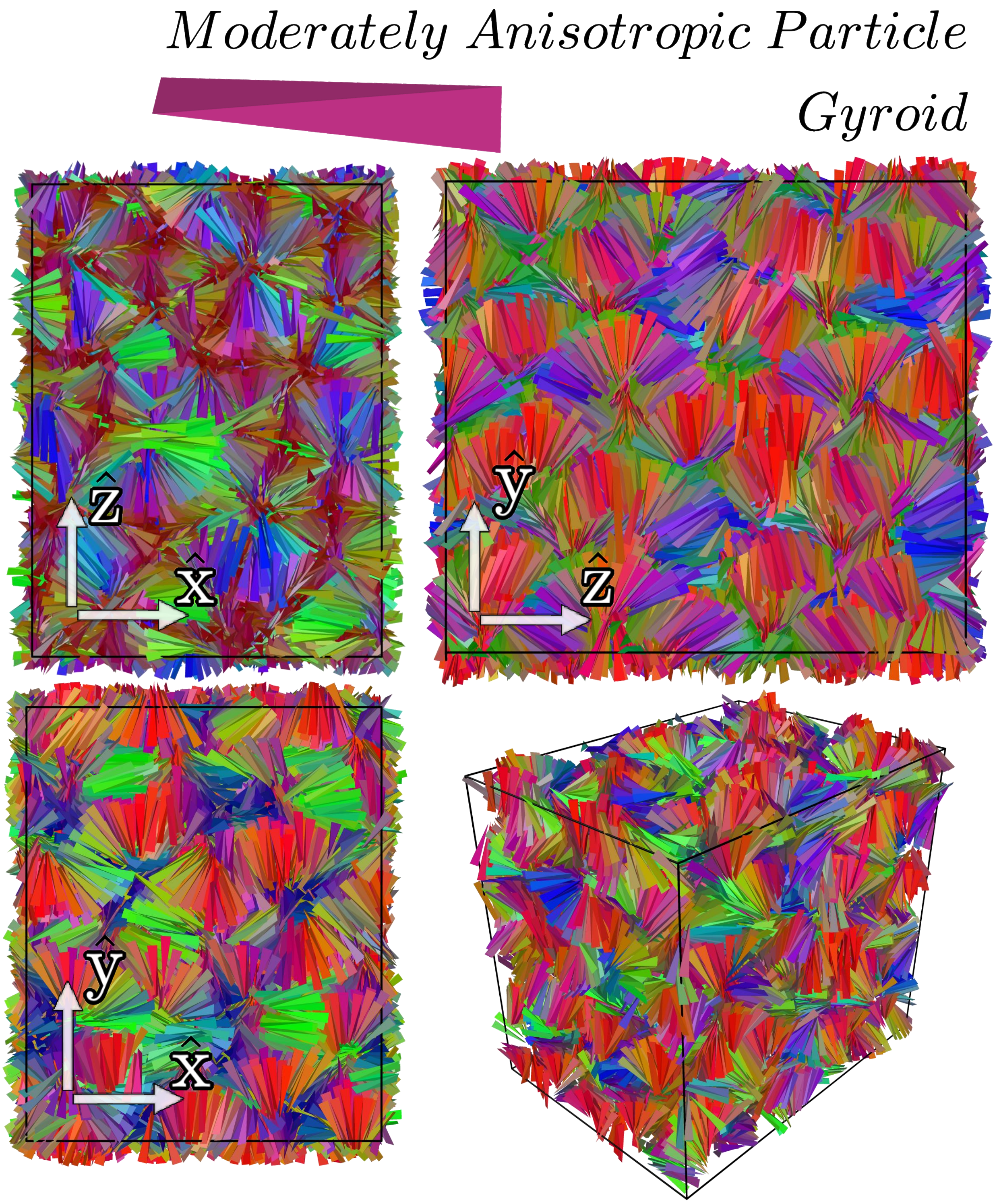

Supplementary Fig. 6. Typical configuration of a system of moderately anisotropic particles in the gyroid smectic phase. The same configuration is shown along the three coordinate axes and in perspective. Particles are colored according to their orientation: green for the x axis, red for the y axis, and blue for the z axis.

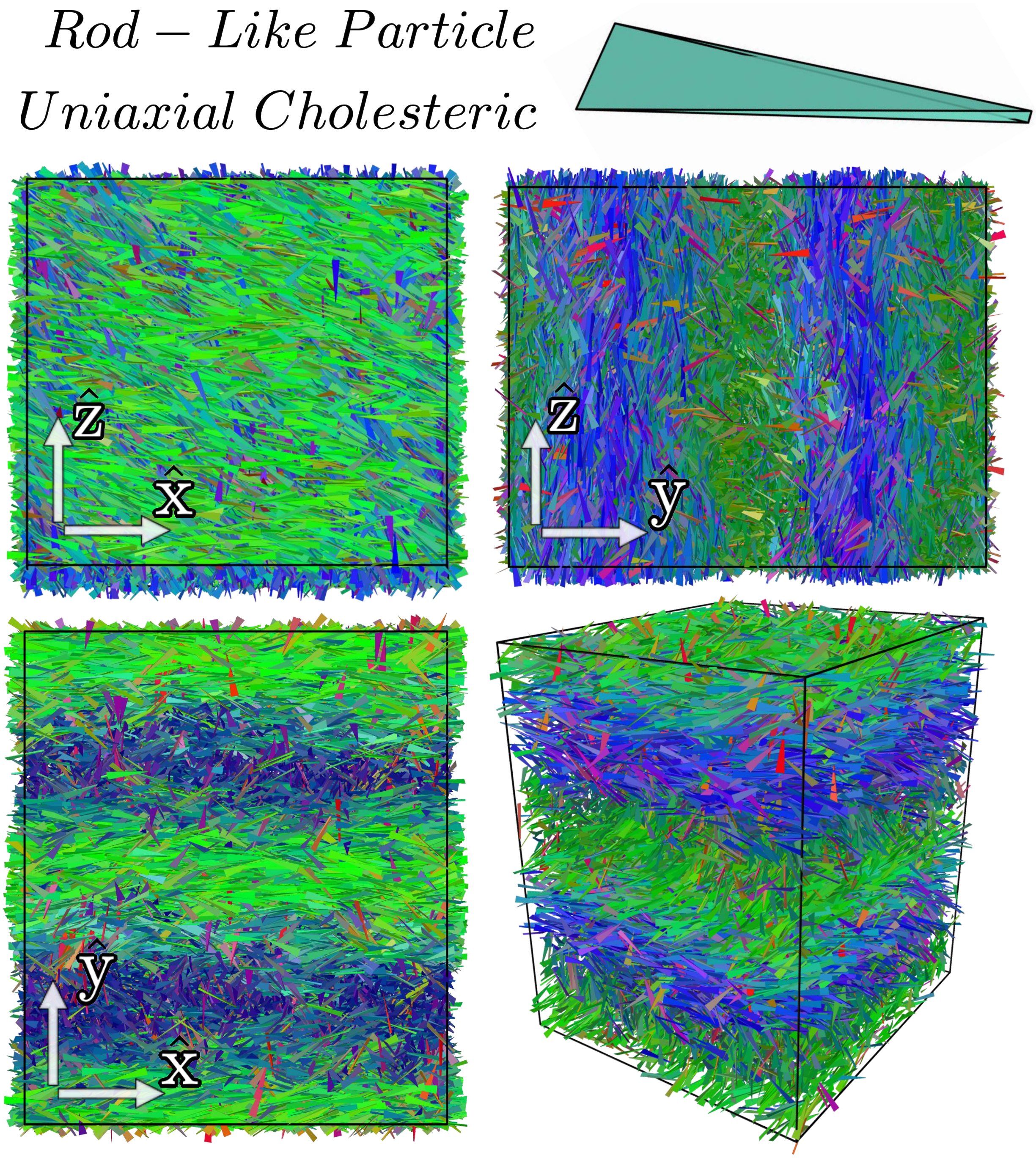

Supplementary Fig. 7. Typical configuration of a system of rod-like particles in the uniaxial cholesteric phase. The same configuration is shown along the three coordinate axes and in perspective. Particles are colored according to their orientation: green for the x axis, red for the y axis, and blue for the z axis.

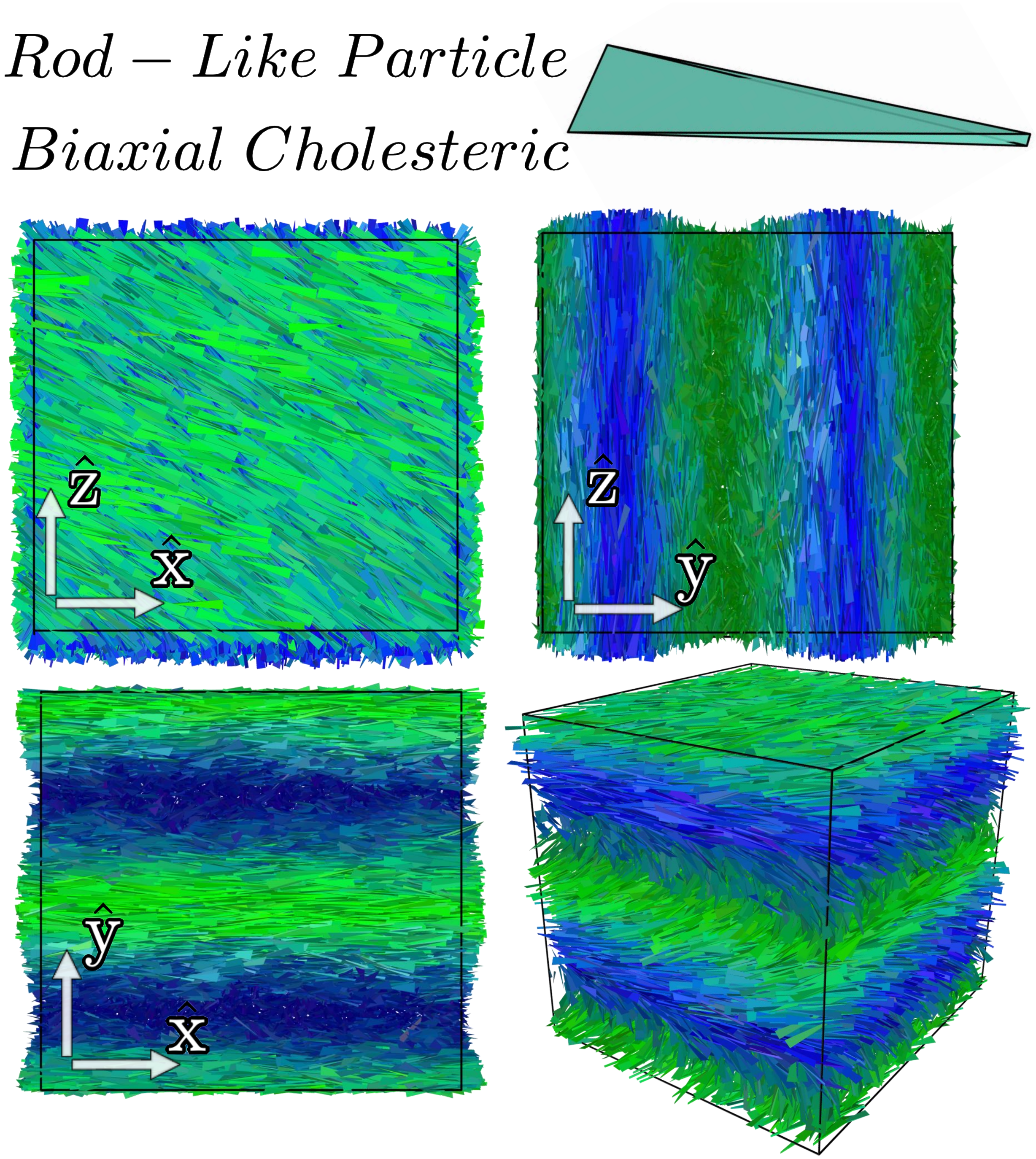

Supplementary Fig. 8. Typical configuration of a system of rod-like particles in the biaxial cholesteric phase. The same configuration is shown along the three coordinate axes and in perspective. Particles are colored according to their orientation: green for the x axis, red for the y axis, and blue for the z axis.

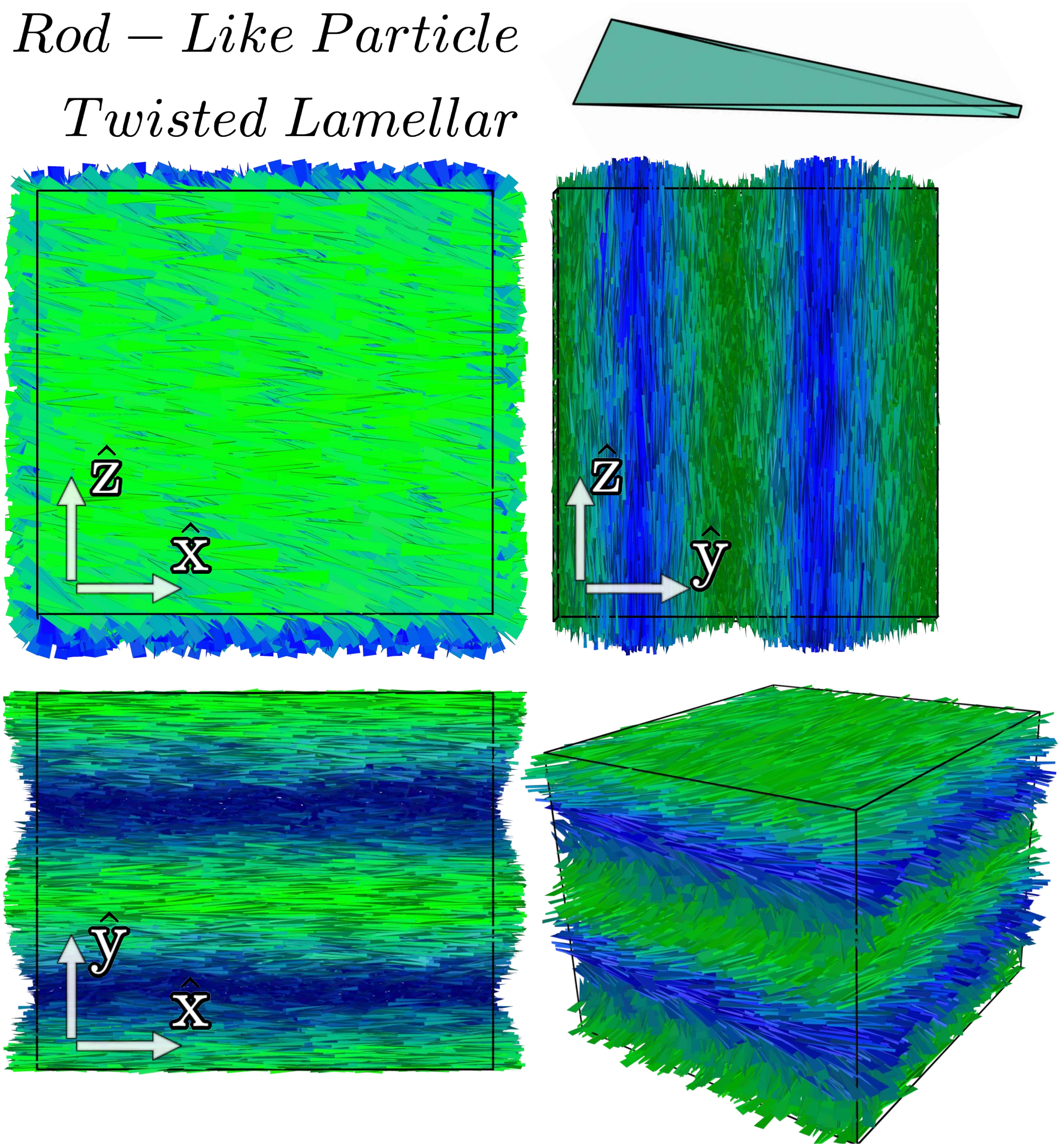

Supplementary Fig. 9. Typical configuration of a system of rod-like particles in the twisted lamellar smectic phase. The same configuration is shown along the three coordinate axes and in perspective. Particles are colored according to their orientation: green for the x axis, red for the y axis, and blue for the z axis.

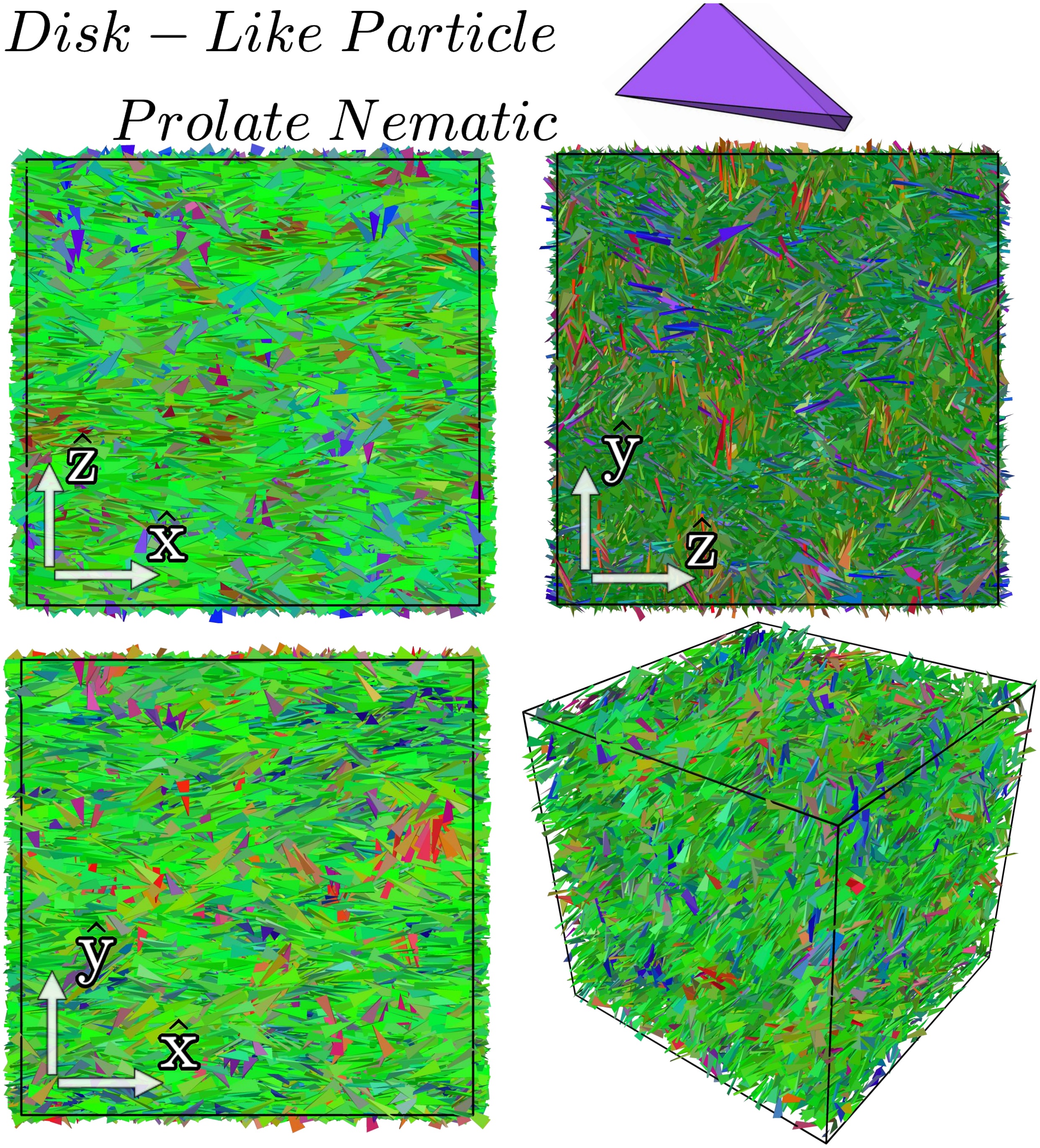

Supplementary Fig. 10. Typical configuration of a system of disk-like particles in the prolate nematic phase. The same configuration is shown along the three coordinate axes and in perspective. Particles are colored according to their orientation: green for the x axis, red for the y axis, and blue for the z axis.

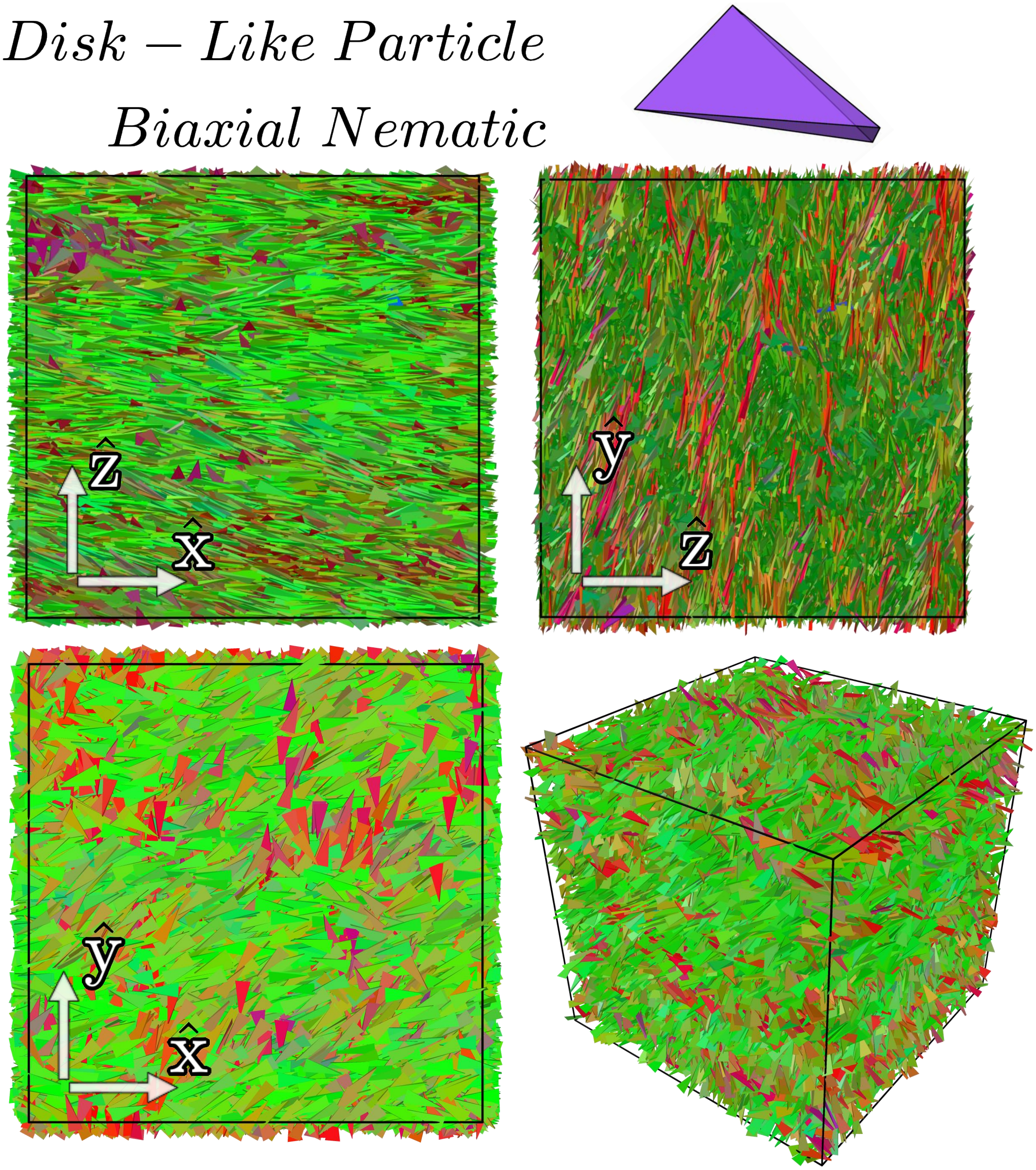

Supplementary Fig. 11. Typical configuration of a system of disk-like particles in the biaxial nematic phase. The same configuration is shown along the three coordinate axes and in perspective. Particles are colored according to their orientation: green for the x axis, red for the y axis, and blue for the z axis.

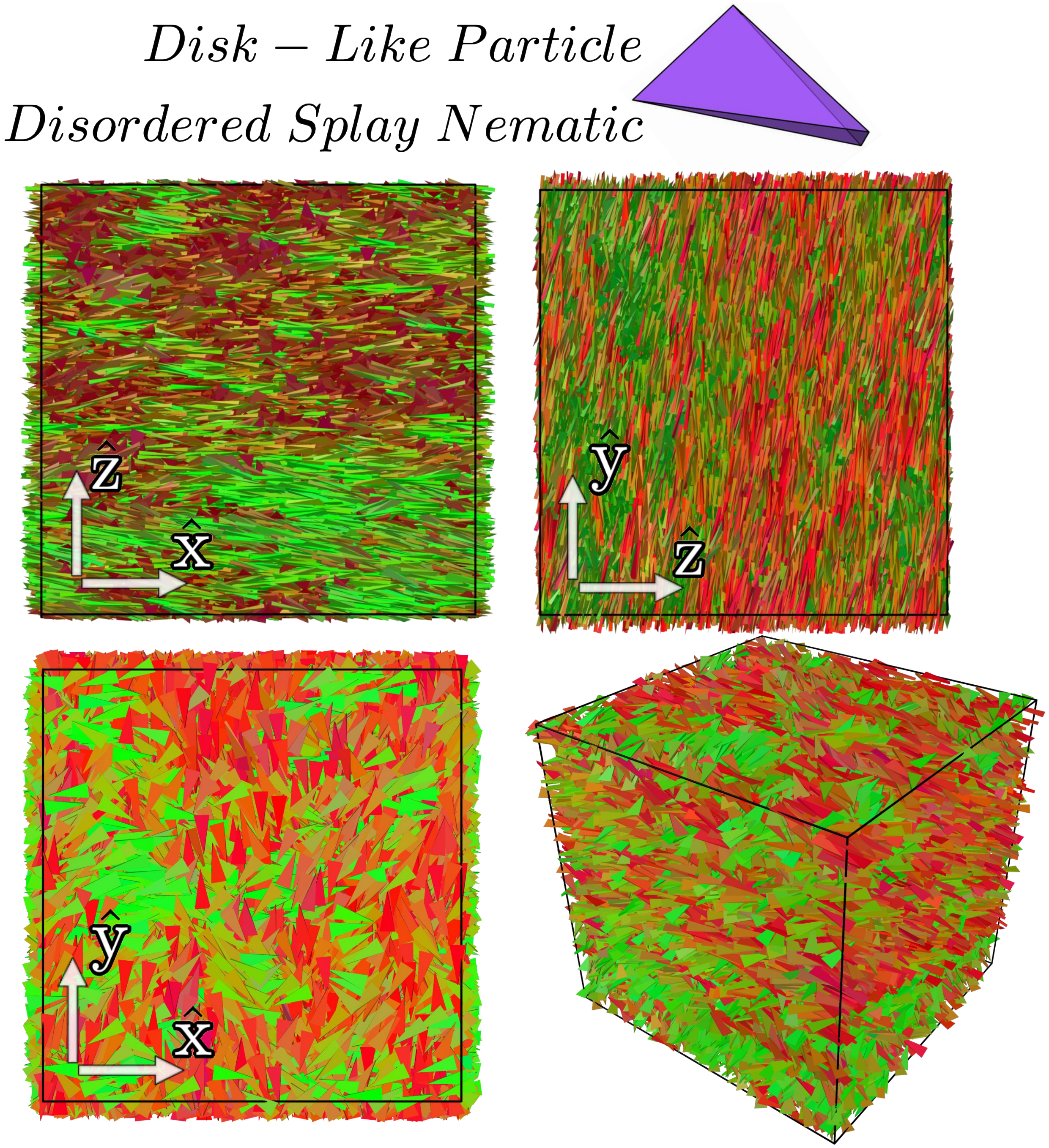

Supplementary Fig. 12. Typical configuration of a system of disk-like particles in the disordered splay nematic phase. The same configuration is shown along the three coordinate axes and in perspective. Particles are colored according to their orientation: green for the x axis, red for the y axis, and blue for the z axis.

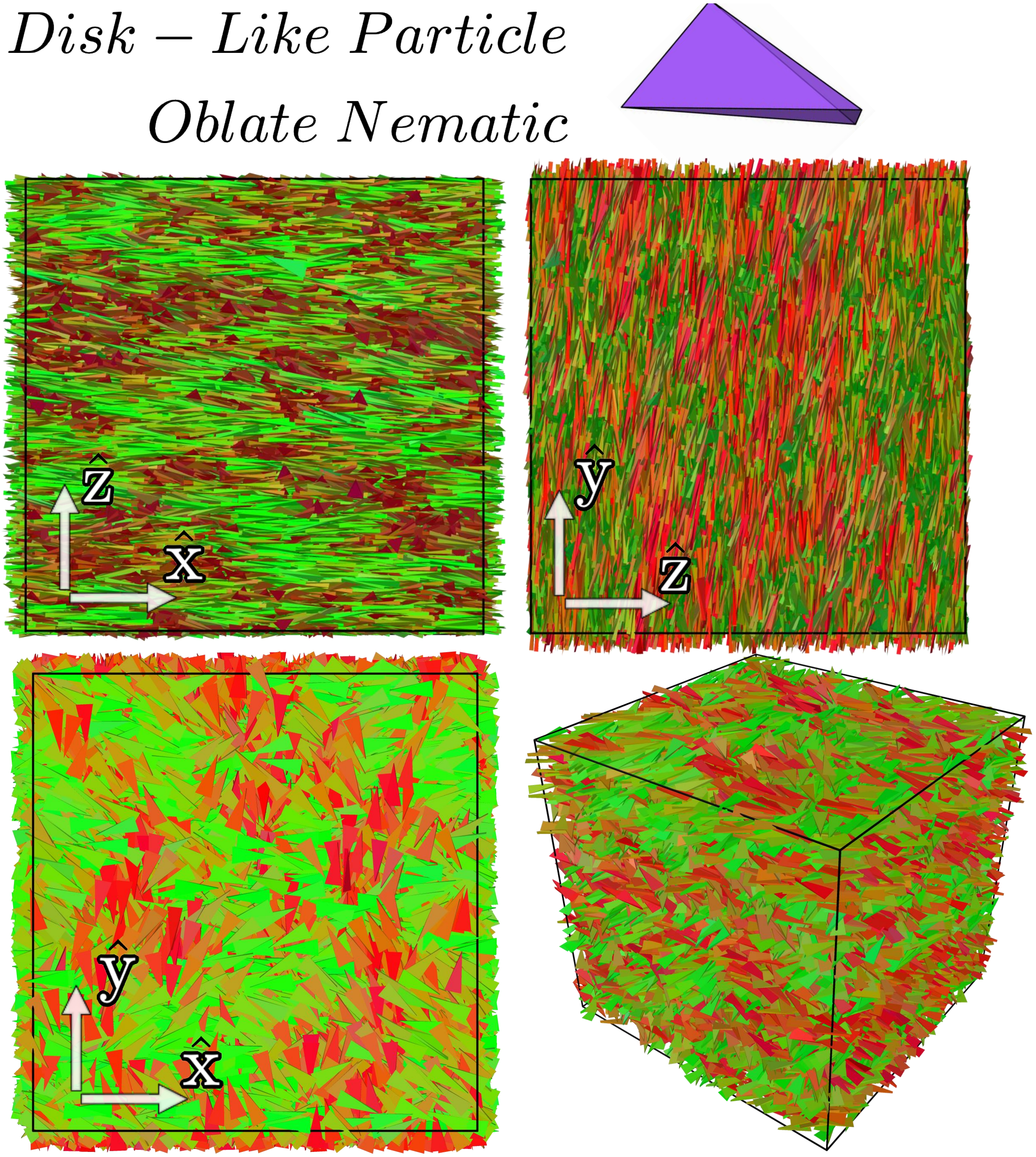

Supplementary Fig. 13. Typical configuration of a system of disk-like particles in the oblate nematic phase. The same configuration is shown along the three coordinate axes and in perspective. Particles are colored according to their orientation: green for the x axis, red for the y axis, and blue for the z axis.

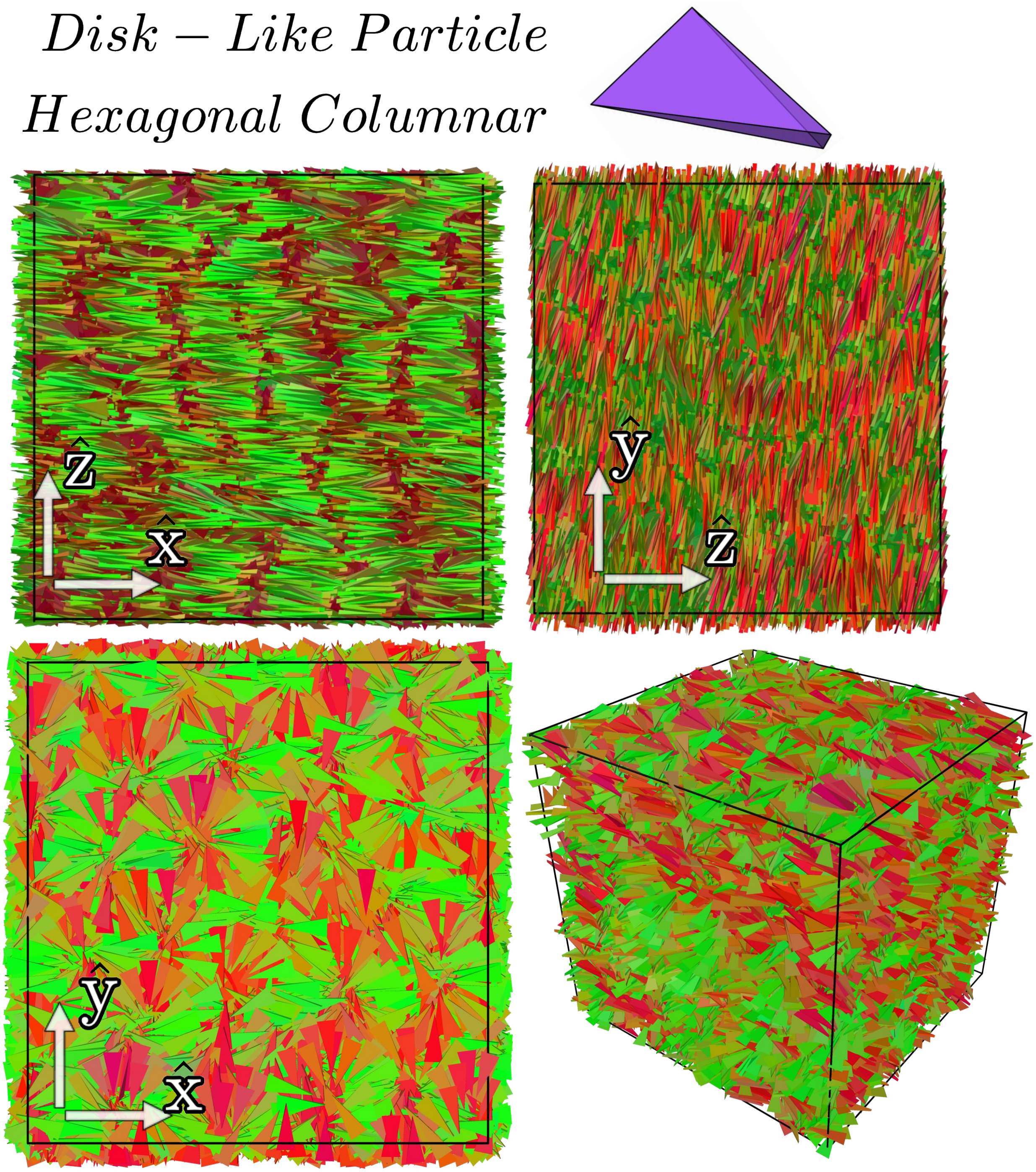

Supplementary Fig. 14. Typical configuration of a system of disk-like particles in the hexagonal columnar smectic phase. The same configuration is shown along the three coordinate axes and in perspective. Particles are colored according to their orientation: green for the x axis, red for the y axis, and blue for the z axis.

[1] J. A. Anderson, J. Glaser, and S. C. Glotzer, Computational Materials Science **173**, 109363 (2020).

[2] N. Chaturvedi and R. D. Kamien, Physical Review E **100**, 022704 (2019).

[3] A. Poniewierski and J. Stecki, Molecular Physics **38**, 1931 (1979).

[4] R. Holyst and A. Poniewierski, Physical Review A **38**, 1527 (1988).

[5] S.-D. Lee, Physical Review A **39**, 3631 (1989).

[6] J. Parsons, Physical Review A **19**, 1225 (1979).

[7] S.-D. Lee, The Journal of chemical physics **87**, 4972 (1987).

[8] J. V. Selinger, Annual Review of Condensed Matter Physics **13**, 49 (2022).

[9] M. P. Rosseto and J. V. Selinger, Physical Review E **101**, 052707 (2020).
