# Supplementary material for: Hierarchical self-assembly of simple hard polyhedra into complex mesophases
Source: Nat Commun. 2025 Dec 4;16:10887. doi: 10.1038/s41467-025-65891-w (PMC12678431; doi:10.1038/s41467-025-65891-w)
Supplement: Supplementary file 2 — Description of Additional Supplementary Files [file 41467_2025_65891_MOESM2_ESM.pdf]

Description of Additional Supplementary Files
Hierarchical Self-assembly of Simple Hard Polyhedra into Complex Mesophases

Rodolfo Subert[1] and Marjolein Dijkstra[1,2]*

[1] *Soft Condensed Matter & Biophysics,*

Debye Institute for Nanomaterials Science, Utrecht University,

Princetonplein 5, 3584 CC Utrecht, The Netherlands. and

[2] *International Institute for Sustainability with Knotted Chiral Meta Matter (SKCM²),*

Hiroshima University, 1-3-1 Kagamiyama,

Higashi-Hiroshima, Hiroshima, 739-8526, Japan

* m.dijkstra@uu.nl

- **Name:** Supplementary Movie 1.

  Description: Typical self-assembly of a spontaneous cholesteric phase upon a pressure quench from the isotropic phase.

- **Name:** Supplementary Movie 2.

  Description: Hyerarchical structure of the hexagonal columnar phase. The movie first shows how tetrahedral particles align along their thin faces to form disks; the disks then stack into columns; finally, the columns, tapered by the disks thin rims, intercalate to produce the hexagonal lattice.

- **Name:** Source_Data.zip.

  Description: A zipped folder containing all numerical data used to generate the three equations of state presented in the main text.

  For each state point, the dataset includes the corresponding values of the two order parameters and the related standard deviation.

  A `README` file within the folder provides a detailed description of the folder structure, file formats, and variable definitions to facilitate data reproduction and analysis.
